# GluN2 Subunit-Dependent Redox Modulation of NMDA Receptor Activation by Homocysteine

**DOI:** 10.3390/biom10101441

**Published:** 2020-10-14

**Authors:** Dmitry A. Sibarov, Sergei I. Boikov, Tatiana V. Karelina, Sergei M. Antonov

**Affiliations:** Sechenov Institute of Evolutionary Physiology and Biochemistry of the Russian Academy of Sciences, Laboratory of Comparative Neurophysiology, 194223 Saint-Petersburg, Russia; dsibarov@gmail.com (D.A.S.); sergei-boickov@mail.ru (S.I.B.); karelina_tanja@mail.ru (T.V.K.)

**Keywords:** homocysteine, thiol, redox modulation, NMDA receptors, glutamate, cysteine, neurons

## Abstract

Homocysteine (HCY) molecule combines distinct pharmacological properties as an agonist of *N*-methyl-d-aspartate receptors (NMDARs) and a reducing agent. Whereas NMDAR activation by HCY was elucidated, whether the redox modulation contributes to its action is unclear. Here, using patch-clamp recording and imaging of intracellular Ca^2+^, we study dithiothreitol (DTT) effects on currents and Ca^2+^ responses activated by HCY through native NMDARs and recombinant diheteromeric GluN1/2A, GluN1/2B, and GluN1/2C receptors. Within a wide range (1–800 μM) of [HCY]s, the concentration–activation relationships for recombinant NMDARs revealed a biphasicness. The high-affinity component obtained between 1 and 100 µM [HCY]s corresponding to the NMDAR activation was not affected by 1 mM DTT. The low-affinity phase observed at [HCY]s above 200 μM probably originated from thiol-dependent redox modulation of NMDARs. The reduction of NMDAR disulfide bonds by either 1 mM DTT or 1 mM HCY decreased GluN1/2A currents activated by HCY. In contrast, HCY-elicited GluN1/2B currents were enhanced due to the remarkable weakening of GluN1/2B desensitization. In fact, cleaving NMDAR disulfide bonds in neurons reversed the HCY-induced Ca^2+^ accumulation, making it dependent on GluN2B- rather than GluN2A-containing NMDARs. Thus, estimated concentrations for the HCY redox effects exceed those in the plasma during intermediate hyperhomocysteinemia but may occur during severe hyperhomocysteinemia.

## 1. Introduction

Homocysteine (HCY) is a thiol-containing amino acid derived from methionine metabolism as a byproduct. The deficit of B_6_ and B_12_ vitamins [1] or mutation in the methylenetetrahydrofolate reductase gene [2] lead to HCY accumulation in the blood plasma and cerebrospinal fluid, which can exceed 100 µM in severe hyperhomocysteinemia [3]. Elevated HCY concentrations contribute to neuronal death in many neurodegenerative diseases like stroke, Alzheimer’s disease, Parkinson’s disease, etc. [4,5]. In vitro, HCY promotes apoptosis in cortical [6,7] and cerebellar [8,9] neurons.

It is well established that HCY activates *N*-methyl-d-aspartate receptors (NMDARs) [10,11] and group I metabotropic receptors [12,13]. The NMDAR activation by HCY and related intracellular calcium accumulation is thought to provide a major contribution to the HCY neurotoxicity [11,14,15]. NMDARs represent tetramer protein complexes composed of two GluN2 subunits, which form an agonist binding site for endogenous amino acids glutamate and HCY, and two GluN1 subunits bearing co-agonist binding sites for glycine [16] or d-serine [17,18]. HCY activates the GluN1/2A subtype of NMDARs with the highest affinity among NMDARs but provokes desensitization of GluN1/2B receptors [19]. This is a pharmacological feature determining the GluN2A-subunit dependence of the HCY neurotoxicity [15,20,21,22,23]. NMDAR competitive antagonist (AP-5) and channel blockers (MK-801 and memantine) prevent HCY-induced neuronal damage both in vitro [7,11] and in vivo [24] during mild and intermediate hyperhomocysteinemia. Despite that the half-maximal concentrations (EC_50_s) for the HCY activation of all NMDAR subtypes are found to be below 100 µM [19,25], further increase of HCY concentrations up to millimolar values results in an additional increase of both currents through NMDARs and subsequent calcium entry into neurons [11]. When the HCY concentration in the plasma reaches 300 µM, which is the level corresponding to the severe hyperhomocysteinemia, non-competitive NMDAR antagonists like MK-801 [11] or memantine [24] are not able to provide the neuroprotection. Under these particular conditions, hyperhomocysteinemia is characterized by disruptions of different organism systems, including the cardiovascular one that may suggest some broad and non-specific effects of HCY. These symptoms may originate from the presence of a thiol group in the HCY molecule, which allows HCY to reduce disulfide bonds due to its higher acid dissociation constant for the thiol group than of cysteine [26,27]. There are some indications that severe hyperhomocysteinemia is accompanied by homocysteinylation of proteins, altering their molecular structure and/or function [28].

NMDARs are subjected to redox regulation. For example, dithiothreitol (DTT) [29], hydrogen sulfide (H_2_S) [30], and glutathione (GSH) [29], which are reducing agents, caused an increase of NMDA and glutamate-activated currents through NMDARs. Considering the high sensitivity of NMDARs to the positive modulation by cleavage of disulfide bonds, we can assume the functional importance of homocysteinylation of cysteines at the extracellular domain of the receptor. The most reactive and functionally valuable disulfide bonds are located in the GluN1 subunits [31,32], common for all NMDARs. Breaking of C744–798 in the glycine binding domain of GluN1 makes the structure of the domain more flexible. It causes a 6-fold decrease of EC_50_ for NMDA binding by GluN2 [31], which considerably increases the channel open probability [32]. Therefore, thiol-containing pharmacological agents like H_2_S, DTT, and GSH can reversibly modify cysteine bonds, which are accessible in NMDAR quaternary structure [29,33]. For instance, H_2_S and DTT cause different effects on currents transferred through GluN1/2A and GluN1/2B receptors in hippocampal slices [33], which could be explained by some difference of functionally essential disulfide bonds like C79–308 and C780–726 in GluN1, and C87–320 in GluN2A, between GluN2A and GluN2B containing NMDARs [34].

HCY stands out against other NMDAR agonists. Its molecule combines multiple pharmacological features being simultaneously the GluN2 glutamate binding site agonist [11] and the redox-active agent. Whereas the effects of HCY low concentrations as a GluN2 agonist were recently described [19,25], no one can exclude some contribution of redox activity in the HCY action on NMDARs at concentrations reached in the plasma during severe hyperhomocysteinemia. Because there is a lack of information on the issue of the redox modulation of NMDARs by HCY, here, we studied the effects of HCY within a wide range of concentrations on native and recombinant diheteromeric NMDARs of GluN1/2A, GluN1/2B, and GluN1/2C subunit compositions. We demonstrated that the HCY effects at concentrations above 100 µM that could be achieved in the plasma during severe hyperhomocysteinemia are GluN2 subunit-dependent on the NMDAR redox status.

## 2. Materials and Methods

### 2.1. Animals and Primary Culture of Cortical Neurons

Animal maintenance and all procedures using animals were approved by the animal care and use committee of Sechenov Institute of Evolutionary Physiology and Biochemistry of the Russian Academy of Sciences (project 16–15-10192, 01.05.2016). Wistar rats (total of 11 pregnant female animals) were sacrificed by 1 min CO_2_ inhalation. Fetuses were used to prepare the primary cultures of rat cortical neurons using conventional procedures described earlier [35,36]. The neuronal culture was supplemented by Neurobasal with B-27 (Gibco-Invitrogen, UK) and grown on glass coverslips coated with poly-d-lysine. Cultures were used for experiments after 12–16 days of in vitro incubation.

### 2.2. HEK293 Cultures and Transfections

Human embryonic kidney (HEK) 293T cells were maintained as previously described [37]. Cells were plated onto glass coverslips covered with poly-d-lysine for 18–24 h, then transiently transfected with a pcDNA1 encoding rat GluN2A, GluN2B, or GluN2C subunits of NMDA receptors, pcDNA3.1 encoding GluN1, and EGFP (1 EGFP: 1 GluN1: 3 GluN2) using FuGene HD reagent (Promega, Madison, WI, USA). Briefly, 50 µL of serum-free medium containing 1 µg total DNA and 2 µL FuGene was added to each 35 mm dish with coverslips. 6–8 h later, the transfection solution was replaced with fresh culture media containing 200 µM d,l-2-amino-5-amino-5-phosphono-valeric acid (d,l-AP-5) and 2 mM Mg^2+^ to prevent NMDA receptor-mediated excitotoxicity. 72 h after transfection, the cells we used for experiments. Mammalian expression vectors were supplied by Dr. J. W. Johnson (University of Pittsburgh, Pittsburgh, PA, USA).

### 2.3. Patch-Clamp Recordings

Whole-cell currents from cultured neurons or HEK293 cells were recorded using a MultiClamp 700B patch-clamp amplifier with Digidata 1440A controlled by pClamp v10.2 software (Molecular Devices). Recordings were low-pass-filtered at 100 Hz. Perfusing solution exchange was performed by BPS-8 fast-perfusion system (ALA Science Inc., Farmingdale, NY, USA) with the tip of the manifold placed 100–200 µm from the recorded cell. The external bathing solution contained (in mM): 140 NaCl; 2.8 KCl; 1.0 CaCl_2_; 10 HEPES, at pH 7.2–7.4, 310 mOsm. The pipette solution contained (in mM): 120 CsF, 10 CsCl, 10 EGTA, and 10 HEPES, 300 mOsm, pH 7.4. Patch-pipettes of 4−6 MΩ were pulled from Sutter BF150–89–10 capillaries. Experiments were performed at 23–25 °C. Both neurons and HEK293 cells were voltage-clamped at −70 mV (holding voltage, V_h_). The liquid junction potential was ~12 mV between the Na^+^-containing bathing solution and the Cs^+^-containing pipette solution. Wherever V_h_ is shown, this value is indicated without a correction for the liquid junction potential. To activate NMDARs, both *N*-methyl-d-aspartate (NMDA) or l-homocysteine (HCY) were always co-applied with 30 µM glycine as a co-agonist.

### 2.4. Calcium Imaging

For loading with Fluo-8, fluorescent Ca^2+^-sensitive dye cortical neurons were incubated in the basic media containing 2 µM Fluo-8 acetoxymethyl ester (Fluo-8 AM) at room temperature for 60 min. The cells were washed out from dye by 20 min incubation in pure basic solution, and coverslips were transferred to a Leica TCS SP5 MP inverted microscope (Leica Microsystems, GmbH, Wetzlar, Germany). The imaging bath was permanently perfused at a flow rate of 1.2 mL/min with the same basic solution as used for patch-clamp recording. Local media exchange was achieved using the fast local-perfusion system, which allowed the rapid application of various compounds. HCY (100 µM) was applied together with 30 µM glycine. Fluo-8 fluorescence was excited with a 488 nm laser and detected at the 510–560 nm spectral range with ~2 s sampling interval (frame 1024 × 1024 pixels, 20× objective). The fluorescence intensities were measured in regions of interest (ROIs) chosen inside of individual neuronal bodies. The intensity of fluorescence in ROIs in the absence of NMDAR agonists was taken as 1.

### 2.5. Data Analysis

Quantitative data are expressed as mean ± SEM. GraphPad Prism software was used for statistical analysis. Student’s two-tailed *t*-test was used to compare groups of measurements. The number of experiments is indicated by ’*n*’. The level of statistical significance was set to *p* < 0.05. Data marked by *, **, ***, or **** are significantly different with *p* < 0.05, *p* < 0.01, *p* < 0.001, or *p* < 0.0001 correspondently.

Approximations of the data were performed with the usage of Origin Pro software. Since increase in HCY concentrations ([HCY]s) resulted in biphasic elevation of transmembrane currents, the concentration-response curves for activation of NMDA receptors with HCY were approximated with biphasic Hill equation I = I_max_ · [*d*/(1 + [L]*^h1^*/[HCY]*^h1^*) + (1 − *d*) / (1 + [H]*^h2^*/[HCY]*^h2^*)], where [L] and [H] are EC_50_s corresponding to low and high concentration components, *h_1_* and *h_2_* are corresponding Hill coefficients, *d* exhibits the fraction of current produced by low concentration component, and I_max_ is the maximal current response. The used biphasic equation gave us the least value of reduced Χ-square root parameter for data fitting compared to the single-component Hill equation.

### 2.6. Reagents

Reagents were obtained from Sigma-Aldrich (St. Louis, MO, USA) except FuGene HD reagent (Promega, Madison, WI, USA) and Neurobasal culture media with B-27 supplement (Gibco, Dublin, Ireland). Ifenprodil (10 µM) was used for GluN2B-specific and ZnCl_2_ (200 nM) for GluN2A-specific inhibition of NMDARs.

## 3. Results

### 3.1. Redox Modulation of HCY-Elicited Currents Is GluN2 Subtype-Specific

**Figure 1:**
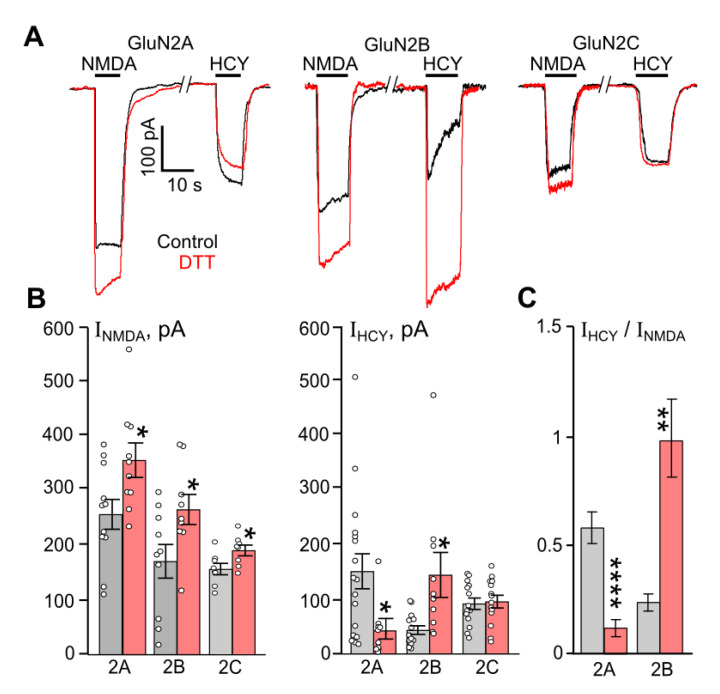
Comparison of DTT effects on currents activated by NMDA and HCY in HEK293 cells expressing recombinant NMDARs. (**A**) Representative current traces recorded at V_h_ = −70 mV in cells expressing either GluN1/2A, GluN1/2B, or GluN1/2C receptors activated by 100 µM NMDA and 100 µM HCY in control (black) and after treatment with 1 mM DTT for 90 s (red). Applications of agonists are shown above the traces by lines. (**B**) Quantitative comparison of steady-state amplitudes of currents elicited by NMDA (I_NMDA_). * Data are significantly different for GluN1/2A (*n* = 11, *p* = 0.02, Student’s *t*-test), GluN1/2B (*n* = 10, *p* = 0.03, Student’s *t*-test), and GluN1/2C (*n* = 7, *p* = 0.03, Student’s *t*-test) receptors. Quantitative comparison of steady-state amplitudes of currents elicited by HCY (I_HCY_). * Data differ significantly for GluN1/2A (*n* = 14, *p* = 0.01, Student’s *t*-test) and GluN1/2B (*n* = 17, *p* = 0.02, Student’s *t*-test) receptors. Mean values ± SEM are plotted. (**C**) Average ratios of the amplitudes of currents (I_HCY_/I_NMDA_) obtained for each cell. **** Data for GluN1/2A receptors differ significantly (*n* = 11, *p* = 0.00008, Student’s *t*-test). ** Data for GluN1/2B receptors differ significantly (*n* = 14, *p* = 0.0017, Student’s *t*-test). For all plots, black columns—the data obtained in control, red columns—the data obtained after treatment with 1 mM DTT. Mean values ± SEM are shown.

As the starting point to evaluate possible dependence of HCY activation of NMDARs on their redox status, the NMDAR currents, evoked by equimolar concentrations (100 µM) of NMDA (I_NMDA_) and HCY (I_HCY_), were compared in HEK293 cells expressing GluN1/2A, GluN1/2B, or GluN1/2C receptors before and after 1 mM DTT treatment (Figure 1A). NMDA-elicited currents were enhanced by DTT for GluN2A- (*n* = 11, *p* = 0.02), GluN2B- (*n* = 10, *p* = 0.03), and GluN1/2C-containing (*n* = 7, *p* = 0.03) NMDARs, which is consistent with previous observations [29,31]. In the case of HCY-activated NMDAR currents recorded in the same cells as for NMDA, the DTT pretreatment caused a considerable increase of current steady-state amplitudes for GluN2B-containing receptors (*n* = 17, *p* = 0.02). In contrast, currents through GluN2A-containing NMDARs were remarkably decreased (*n* = 14, *p* = 0.01), while a lack of effect was found for GluN1/2C NMDARs (Figure 1B). Therefore, the DTT pretreatment increased amplitudes of both HCY- and NMDA-activated currents for NMDARs containing GluN2B subunits. However, DTT induced opposite effects on NMDAR currents activated by HCY and NMDA when tested on NMDARs containing GluN2A subunits.

To improve a statistical comparison of DTT effects and eliminate HEK293 cell variability concerning NMDAR expressions, in each pair of NMDA and HCY applications, the steady-state amplitudes of NMDAR currents activated by 100 µM NMDA were used as a reference current, presumably corresponding to saturated receptor activation. We, therefore, normalized the steady-state amplitude achieved during the HCY application (I_HCY_) by the steady-state amplitude achieved during the NMDA application (I_NMDA_). This allowed presenting HCY-elicited currents as a fraction of NMDA-activated current corresponding to saturated receptor activation (Figure 1C). The ratio (I_HCY_/I_NMDA_) values exhibited less dispersion and strengthen the statistical significance of DTT effects on HCY-elicited currents for both GluN2A (*n* = 11, *p* = 0.00008) and GluN2B (*n* = 14, *p* = 0.0017) containing NMDARs (Figure 1C).

**Figure 2:**
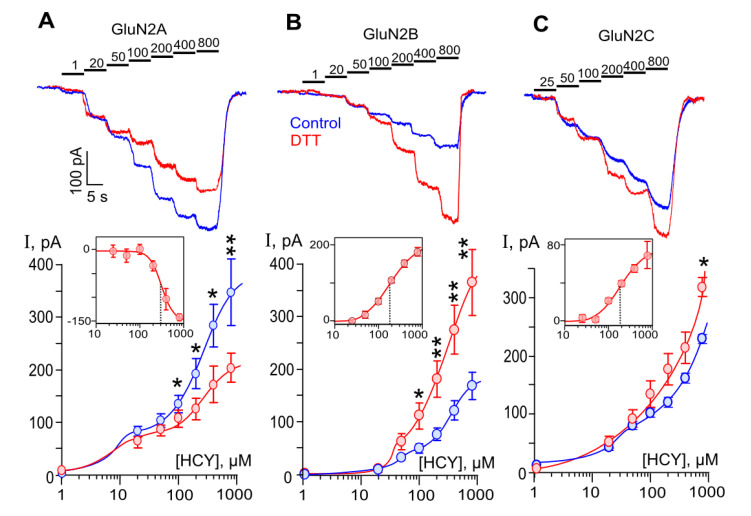
DTT effects on currents activated by HCY through recombinant NMDARs. Concentration dependence of HCY-elicited currents in HEK293 cells expressing GluN1/2A, GluN1/2B, or GluN1/2C NMDA receptors, (**A**–**C**), correspondently. (Top panels) Currents elicited by HCY concentrations (indicated above the records in µM) in control (blue) and after 90 s treatment with 1 mM DTT (red) recorded at V_h_ = −70 mV. (Bottom panels) Concentration-response curves obtained in control (blue) and DTT-treated cells (red) expressing GluN1/2A (*n* = 8), GluN1/2B (*n* = 9), and GluN1/2C (*n* = 15) NMDARs. Data are significantly different from control by Student’s *t*-test in GluN1/2A (*p* = 0.03 *, 0.01 *, 0.01 *, 0.004 ** for [HCY]s of 100, 200, 400, 800 µM), GluN1/2B (*p* = 0.01 *, 0.007 **, 0.002 **, 0.001 ** for [HCY]s of 100, 200, 400, 800 µM), and GluN1/2C (*p* = 0.02 * for [HCY] of 800 µM) NMDARs. Mean values ± SEM are shown. The curves are fitted using biphasic Hill equation. The inserts show the difference between currents obtained at the same [HCY]s by subtracting currents recorded in control from currents recorded after DTT treatment. The curves are fitted using monophasic Hill equation with the following parameters: IC_50_ = 308 ± 31 µM, *h* = 3.3 ± 0.8 (*n* = 5); EC_50_ = 180 ± 7 µM, *h* = 1.4 ± 0.2 (*n* = 6); EC_50_ = 185 ± 49 µM, *h* = 1.6 ± 0.6 (*n* = 6), for GluN1/2A, GluN1/2B, and GluN1/2C NMDARs, correspondently. Mean values ± SEM are shown.

We further studied HCY-activated currents within a wide range of HCY concentrations from 1 µM to 800 µM before and after 1 mM DTT treatment. Sequential stepwise HCY applications with an increment concentration caused currents with subsequently increased amplitudes for all GluN1/2A (Figure 2A), GluN1/2B (Figure 2B), and GluN1/2C (Figure 2C) receptors under study. The concentration-response curves of HCY-elicited currents for GluN1/2A (Figure 2A), GluN1/2B (Figure 2B), and GluN1/2C (Figure 2C) NMDARs are shown below the current traces.

In general, for all three types of NMDARs, the dependence of the amplitude of currents on HCY concentration appeared to be biphasic under control conditions. Previously, we demonstrated that activation of these receptors within the range of HCY concentrations from 1 to 200 µM reaches saturation and could be well fit with the Hill equation [19,25]. Here, in experiments on GluN1/2A receptors using up to 800 µM HCY, we observed biphasic concentration dependence of amplitudes of HCY-activated currents, in which an initial receptor activation with EC_50_ < 10 µM [19] was followed by the saturation at 50–100 µM and further additional current increase at [HCY]s ≥ 200 µM. As a result, the current concentration dependence was better fitted with the biphasic Hill equation than a monophasic one (Figure 2A). Notably, the amplitudes of currents did not reach saturation even at [HCY] = 800 µM. Similar biphasic concentration dependence of currents was found for GluN1/2B and GluN1/2C NMDARs unless the initial portion of curves was characterized by EC_50_s of about 50 µM [19,25] that caused the break in the curves to be less obvious. It should be noted that the second branch of curves was found at similar [HCY]s for all three types of NMDARs, suggesting similar mechanisms of HCY action. Because HCY molecules contain a thiol group, we assume that possible self-potentiation of HCY-activated currents by HCY interaction with NMDAR redox sites may contribute to this reaction. We did not test [HCY]s higher than 800 µM in this type of experiments because they substantially exceeded physiological values observed in hyperhomocysteinemia.

To evaluate how the redox status of NMDA receptors would affect the HCY action, similar measurements to those described above were performed after treatments of HEK293 cells with 1 mM DTT for 90 s, which presumably should cause reductions of a majority of NMDAR disulfide bonds. In contrast to the DTT effects on NMDA-activated currents, the pretreatment with DTT caused a decrease of currents activated by HCY through GluN1/2A receptors (Figure 2A). In agreement to its action on NMDA-activated currents, DTT increased HCY-activated currents through GluN1/2B (Figure 2B) and GluN1/2C (Figure 2C) receptors. The DTT effects depended on [HCY] since any influence on currents activated by HCY below 100 µM was not found. In addition, even at 800 µM HCY, the enhancement of currents through GluN1/2C receptors was still rather moderate (Figure 2C).

The difference between currents obtained in the same [HCY]s before and after DTT treatment (Figure 2, inserts) is likely to demonstrate a pure contribution of the redox modulation NMDARs in the HCY action. The calculated concentration dependence of this effect was well fitted with the Hill equation. Estimated values for the half-maximal concentrations were about 180 µM for GluN1/2B and GluN1/2C NMDARs (Figure 2, inserts B,C) and 308 µM for GluN1/2A receptors (Figure 2, insert A). These values substantially exceed the EC_50_ values for the HCY activation of NMDARs. The Hill coefficient of 1.4–1.6 obtained for GluN1/2B and GluN1/2C suggests a cooperative interaction of two redox sites. The value of Hill coefficient > 3 may argue for the impact of additional sites for the redox modulation for GluN1/2A receptors.

Our experiments demonstrated that the DTT effects are most pronounced in GluN1/2B receptors, which, as known, are desensitized when activated by HCY [19]. The DTT pretreatment increases HCY-activated currents through GluN1/2B receptors in a much greater extent than NMDA-activated currents (Figure 1A), so that the amplitudes of HCY- and NMDA-evoked currents became similar (Figure 1C). This allows us to suggest that the cleavage of disulfide bonds could abolish GluN1/2B receptor desensitization, and redox regulation could contribute to the HCY effects at a concentration above 100 µM.

### 3.2. Redox Modulation of HCY-Induced Desensitization of Native NMDARs

**Figure 3:**
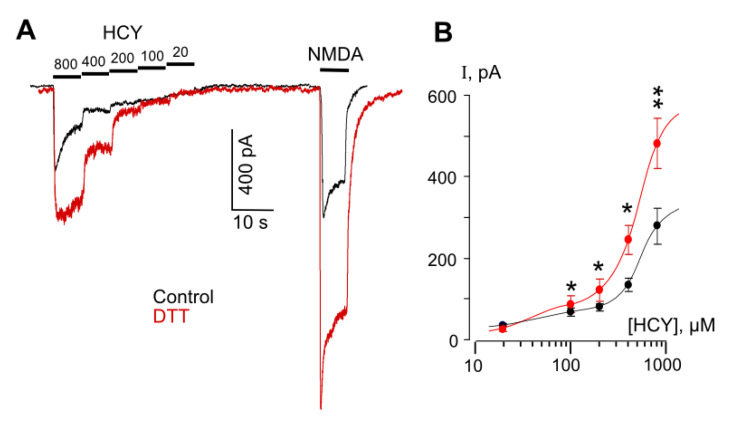
DTT enhances HCY- and NMDA-elicited currents in cortical neurons. (**A**) Representative currents recorded at V_h_ = −70 mV in neurons elicited by decreasing HCY concentrations ([HCY]) and by 100 µM NMDA in control (black) and after treatments with 1 mM DTT for 90 s (red). Applications of agonists and values of [HCY]s are shown (in µM) above the traces. (**B**) Concentration–activation relationships obtained under control conditions (black) and after treatment with DTT (red). Mean values ± SEM are shown (*n* = 8). Data are significantly different from control *p* = 0.02 *, 0.01 *, 0.01 *, 0.003 ** for [HCY]s of 100, 200, 400, and 800 µM, Student’s *t*-test. The curves are fitted using biphasic Hill equation.

**Figure 4:**
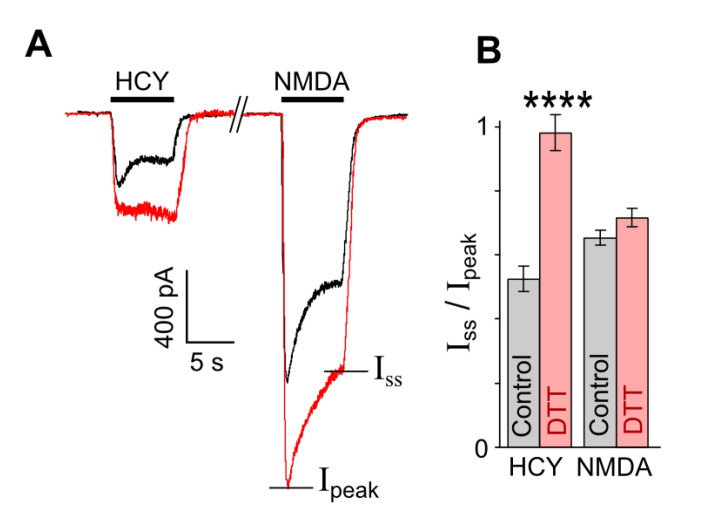
DTT prevents desensitization of currents elicited by HCY, but not by NMDA. (**A**) Representative traces of currents recorded at V_h_ = −70 mV, elicited in neurons by 200 µM HCY and 200 µM NMDA in control (black) and after treatment with 1 mM DTT for 90 s (red). (**B**) Mean steady-state to peak (I_ss_/I_peak_) ratios obtained for HCY- and NMDA-elicited currents before and after treatment with DTT. **** Data are significantly different from control *n* = 8, *p* = 0.00007, Student’s *t*-test.

In order to obtain more evidence in favor of our assumption that the HCY redox action may abolish the GluN1/2B receptor desensitization, experiments were performed on native receptors of cortical neurons. It is well established that cortical neurons express GluN1, GluN2A, and GluN2B subunits, which form diheteromeric and triheteromeric NMDARs [38,39,40,41]. The co-existence of a mixture of different NMDARs in the plasma membrane of cortical neurons predicts a more complex effect of DTT than the effects found in pure receptor populations. Indeed, NMDAR currents activated by HCY in neurons which are expected to be transferred by GluN2A-containing receptors because of the desensitization of GluN2B-containing NMDARs [19] and might be depressed by the DTT pretreatment (Figure 2A), demonstrated a considerable increase of amplitudes in a wide range of [HCY]s (Figure 3A), whereas the DTT effect on NMDA-activated currents agreed to those found on recombinant receptors (Figure 2). In addition, as for the recombinant receptors, the concentration activation curves for HCY in neurons were biphasic, and DTT increased currents, suggesting a major contribution of GluN2B-containing NMDARs in their amplitudes (Figure 3B). Furthermore, in neurons, 100 µM HCY-elicited currents were characterized by >50% desensitization (Figure 4A). The DTT pretreatment abolished the desensitization of HCY-activated currents (Figure 3A and Figure 4A) (*p* = 0.00007, *n* = 8, Student’s *t*-test), but did not influence the desensitization of NMDA-elicited currents (Figure 4A,B). These experiments support our conclusion that changes of GluN1/2B receptor redox status either by the DTT pretreatment or during the action of HCY concentrations ≥ 200 µM may prevent their HCY ligand-dependent desensitization.

### 3.3. The Contribution of GluN2A and GluN2B-Containing NMDARs to HCY Redox Effects in Neurons

**Figure 5:**
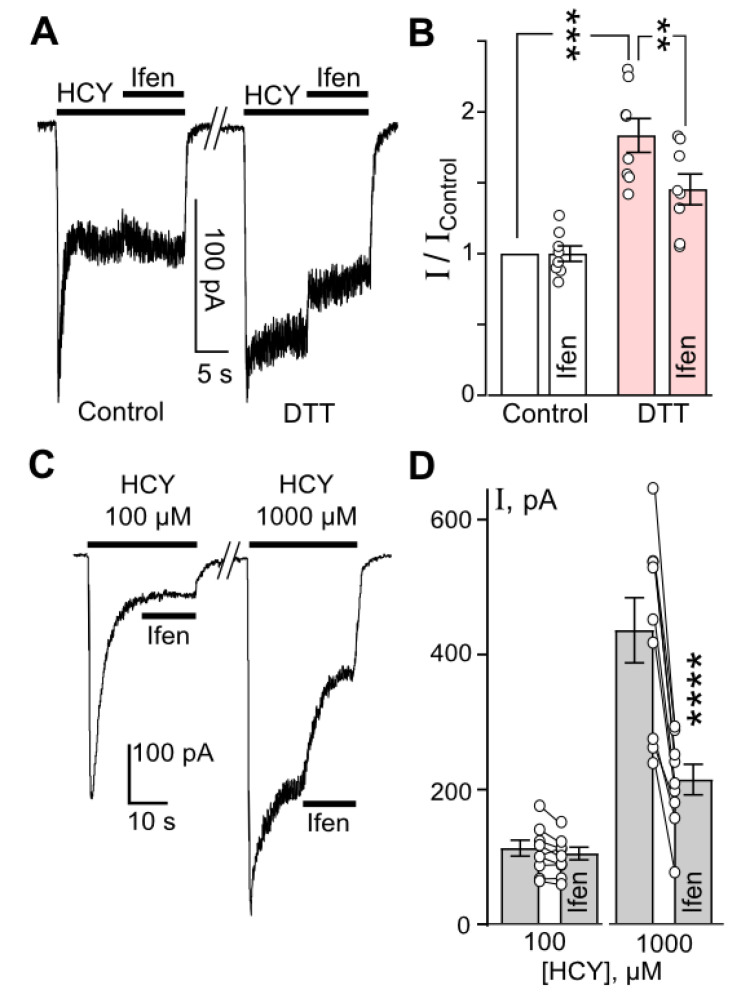
Cleavage of disulfide bonds by both DTT or HCY enhances GluN1/2B contribution to HCY-elicited currents. (**A**) Representative current traces recorded at V_h_ = −70 mV in neurons activated by 100 µM HCY in control and after 1 mM DTT treatment. When current amplitudes reached the steady state, 10 µM ifenprodil (Ifen) was applied. The protocol of applications is shown above the traces. (**B**) Quantitative comparison of Ifen inhibition of HCY-activated currents obtained in control and after DTT treatment. Ordinate represents the current values obtained at steady state (I) normalized to the value obtained in control (I_Control_) without Ifen or DTT treatment. DTT caused a significant increase in HCY-elicited currents (***, *n* = 8, *p* = 0.0002, Student’s *t*-test). Ifenprodil significantly inhibited HCY-evoked currents after DTT treatment (**, *n* = 8, *p* = 0.0022, Student’s *t*-test). (**C**) Representative current traces recorded at V_h_ = −70 mV in neurons activated by 100 or 1000 µM HCY. When currents reached the steady state, Ifen was applied. The protocol of applications is shown above the traces. (**D**) Quantitative comparison of Ifen inhibition of HCY-activated currents. Currents activated by 1000 µM HCY were significantly inhibited by Ifen (****, *n* = 8, *p* = 0.00008, Student’s *t*-test), but not the currents activated by 100 µM HCY (*n* = 8, *p* = 0.24). Circles connected by lines indicate paired measurements in a single experiment.

At this point, it seems appropriate to use GluN2 subtype-specific inhibitors to study the contribution of GluN2A and GluN2B subunits to the redox regulation of HCY effects in neurons. In control neurons, the steady-state currents evoked by HCY were not inhibited by GluN2B antagonist ifenprodil (10 µM) (Figure 5A). Pretreatment of neurons with 1 mM DTT for 90 s caused a significant increase (about 2-fold) of HCY-elicited steady-state currents (*n* = 8, *p* = 0.0002). After DTT treatment, the HCY-activated currents were partially inhibited by ifenprodil (*n* = 8, *p* = 0.0022) (Figure 5B). An appearance of ifenprodil-sensitive component of HCY-induced currents after the treatment with DTT could be explained by the increased contribution of GluN2B-containing NMDARs to HCY-activated currents.

To verify that HCY itself can induce DTT-like effects in NMDARs, we compared ifenprodil action on currents activated by either 100 or 1000 µM HCY (Figure 5C). Whereas ifenprodil did not inhibit currents elicited by 100 µM HCY (Figure 5A,C), this GluN2B-selective antagonist of NMDARs caused a pronounced (of about 50%) inhibition of currents activated by 1000 µM HCY (Figure 5C,D). Therefore, the fraction of NMDAR currents transferred by GluN2B-containing receptors considerably increases when currents are activated by 1000 µM HCY in comparison with currents activated by 100 µM HCY. This observation supports our conclusion that the cleavage of disulfide bonds both by 1 mM DTT and 1 mM HCY can induce similar gain of GluN2B-dependent HCY-activated currents, which occurs due to abolishing agonist-dependent desensitization of GluN2B-containing NMDARs.

**Figure 6:**
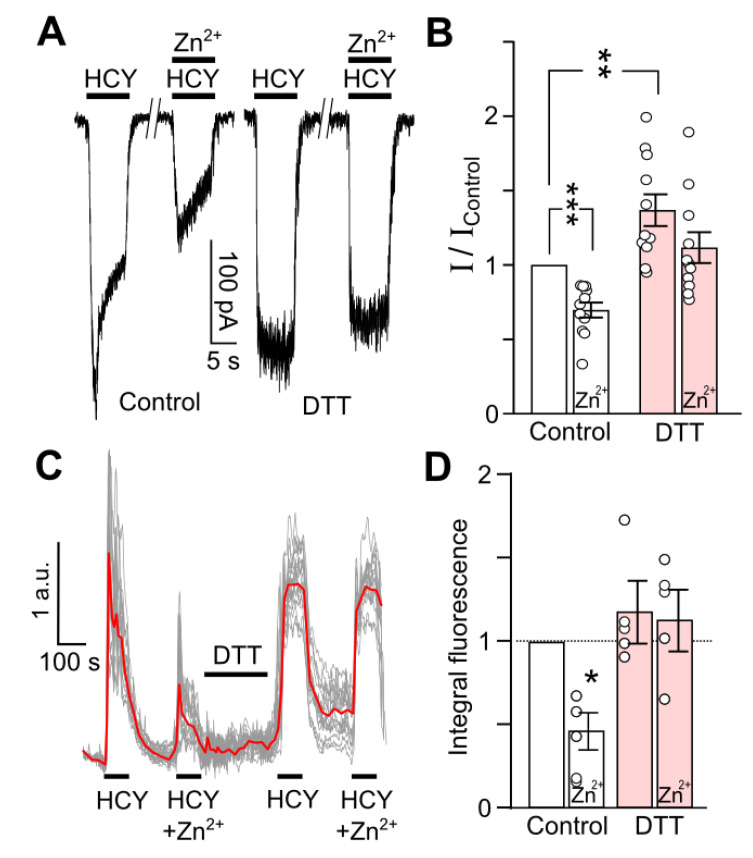
GluN2A-containing NMDAR contribution to HCY-activated currents and intracellular accumulation decreases after DTT treatment. (**A**) Representative traces of currents activated by 100 µM HCY or by 100 µM HCY + 200 nM Zn^2+^ recorded at V_h_ = −70 mV in neurons in control and after 90 s treatment with 1 mM DTT. The protocol of applications is shown above the traces. (**B**) Quantitative comparison of Zn^2+^ inhibition of HCY-activated currents obtained in control and after DTT treatment. DTT significantly increases HCY-elicited currents (**, *n* = 11, *p* = 0.004, Student’s *t*-test). Zn^2+^ significantly inhibits HCY-evoked currents only in control (***, *n* = 11, *p* = 0.0001, Student’s *t*-test). Ordinate represents the current values obtained at steady state (I) normalized to the value obtained in control (I_Control_) without Zn^2+^ or DTT treatment. (**C**) Fluorescent Ca^2+^ responses induced by 100 µM HCY or 100 µM HCY + 200 nM Zn^2+^ in control and after DTT treatment of neurons loaded with Fluo−8. Traces represent an overlay of individual neuronal responses (gray) and average response (red). The protocol of applications is shown above the traces. (**D**) Quantitative comparison of mean integral Ca^2+^-induced fluorescence observed during HCY applications obtained from four coverslips with neuronal cultures. Ordinate represents the values of integral Ca^2+^ entry normalized to control response obtained without Zn^2+^ or DTT treatment. Zn^2+^ significantly inhibits HCY-evoked Ca^2+^ responses only in control (*, *n* = 4, *p* = 0.02, Student’s *t*-test), but not after DTT treatment.

We further focused on the contribution of GluN2A-containing NMDARs to HCY-elicited currents, since these receptors provide a major contribution to HCY-induced neurotoxicity. Zinc (200 nM), used as a specific GluN2A-subunit antagonist [42,43], caused a remarkable (*n* = 11, *p* = 0.0001) inhibition of current elicited by 100 µM HCY (Figure 6A,B). However, after DTT treatment, zinc inhibition of the currents was weakened (Figure 6B). This observation favors the suggestion that DTT potentiates HCY-induced response of GluN2B-, but not GluN2A-containing NMDARs.

The cytosolic-free Ca^2+^ increase by HCY governs its neurotoxicity. To confirm our electrophysiological data, we further performed optical imaging of intracellular Ca^2+^ to evaluate the DTT effects on a population of neurons. In control cortical neurons, the Ca^2+^ responses to 100 µM HCY were effectively suppressed by Zn^2+^ (*n* = 4, *p* = 0.02). (Figure 6C,D), which specifically inhibit GluN2A-containing NMDARs. This suggests that under control conditions, the HCY-induced Ca^2+^ entry was predominantly GluN2A-subunit-dependent. DTT pretreatment completely prevented Zn^2+^ inhibition of HCY-induced Ca^2+^ responses, which speaks in favor of the GluN2B-mediated Ca^2+^ entry after the NMDAR redox modification. This observation coincides well with the conclusion that DTT suppresses HCY-induced GluN1/GluN2A and enhances GluN1/GluN2B-mediated transmembrane currents.

## 4. Discussion

We demonstrated here that the cleavage of disulfide bonds by DTT modulates NMDA- and HCY-elicited NMDAR currents in different ways. DTT enhanced amplitudes of NMDA-activated currents for GluN1/2A, GluN1/2B, and GluN1/2C receptors and did not affect the NMDAR desensitization. In HCY-elicited currents, DTT augmented currents through GluN1/2B and GluN1/2C NMDARs, while GluN1/2A receptor currents were even reduced. Under control conditions, HCY-evoked GluN1/2B currents exhibited pronounced desensitization [19], which almost disappeared after cleaving disulfide bonds with 1 mM DTT or 1 mM HCY. In contrast, HCY-elicited GluN1/2A currents decreased after DTT treatment. Therefore, the redox status of NMDARs may govern the contribution of GluN2A and GluN2B subunit-containing receptors to HCY-caused neurotoxicity, which is an unexpected conclusion. This observation can provide some clues as to why treatment with reducing reagents like hydrogen sulfide (H_2_S) rescues NMDAR expressing neuroblastoma cells [44], neurons [45], and prevents behavioral alterations [46] in hyperhomocysteinemia.

### 4.1. Molecular Basis of Redox Agent Effect on NMDARs

The structural basis of redox action on NMDARs is well established. The observed diverse HCY effects on NMDARs of different subunit compositions may originate from a difference in molecular locations of redox sites in the structure of GluN1/2A and GluN1/2B receptors [29,31]. The most functionally essential pairs of cysteines 744–798 [31] and 780–726 [32] are located in the GluN1 subunits common for all NMDARs. It is known, however, that distinct conformational states of GluN1 in the structure of GluN1/2A and GluN1/2B receptors alter the access of reducing agents to disulfide bonds [47]. For example, enhancement of glutamate-elicited GluN1/2A currents can be achieved by the cleavage of any of the following bonds: GluN1 780–726 [32], 744–798 [31], 79–308 [34], and GluN2A 87–320 [34]. In contradiction, only a reduction of 744–798 [31] augments GluN1/2B currents. As a result, in GluN1/2B receptors, redox agents enlarge the frequency of openings, while in GluN1/2A receptors, these thiol-containing compounds also increase the open time of receptors [47], presumably due to interaction with additional redox sites existing in the GluN2A subunit of NMDARs.

The thiol-containing agents acting from the outside of cells can interact disulfides at the N-terminal domain of NMDARs. DTT represents a traditional, widely used reagent to study the redox regulation of NMDAR functional properties. In all NMDAR subunit compositions, DTT increases currents elicited by glutamate or NMDA [29,31]. Unlike DTT, endogenous reducing agents, including H_2_S, HCY, or glutathione, when causing the cleavage of disulfide bonds, stay bound to thiol groups of NMDARs. Hence, *s*-persulfidation (*s*-sulfhydration) with H_2_S results in the conversion of thiol groups to perthiols (persulfides), causing enhanced GluN1/2A and suppressed GluN1/2B charge transfer [33]. Conversely, DTT as a sulfhydryl-reducing agent diminishes H_2_S effects on NMDARs [33].

HCY can reduce disulfide bonds due to its higher acid dissociation constant for the thiol group as of cysteine [26,27]. Thus, HCY cleaves accessible cysteine disulfide bonds and binds to cysteine residues (*s*-homocysteinylation), altering protein molecular structure and/or function [28]. Elevated [HCY] in animal hyperhomocysteinemia may lead to *s*-homocysteinylation of most reactive free cysteine residues and intramolecular disulfide bonds of proteins [48].

### 4.2. HCY as NMDAR Agonist and Reducing Agent

We hypothesized that HCY at concentrations above 200 µM acts as the reducing agent for NMDARs. There are no quantitative data on the subject of NMDAR *s*-homocysteinylation. However, there are some clues concerning a reducing [HCY], since many other proteins reach saturated *s*-homocysteinylation at [HCY] ~100–140 µM [27,49], which is in order of magnitude higher than EC_50_ for GluN1/2A receptor activation [19]. In agreement, similar EC_50_ values for the redox modulation by HCY (180 µM) of NMDARs were obtained in our experiments. We suppose that for GluN1/2A NMDARs, the biphasic concentration–activation relationship allows a pure distinction between the HCY agonist effect (EC_50_ ~ 10 µM) and the HCY-positive redox modulation (EC_50_ > 300 µM) of NMDARs. For other NMDAR subunit compositions (GluN1/2B and GluN1/2C), the EC_50_s for HCY as an agonist of NMDARs are about 50 µM, and EC_50_s for the HCY redox modulation are ~180 µM. The short gap between EC_50_ values of two distinct modes of HCY effects makes a biphasicness of the concentration–activation curves less articulated.

The modification of NMDAR redox status by DTT caused remarkable modulation of HCY-activated currents only when [HCY]s were >100–200 µM, and the statistical significance of DTT effects tended to increase with the rise of [HCY]s. It is clear that the efficacy of HCY as a reducing agent expectably augments with the [HCY] increase. It should be noted that the sign (negative or positive) of DTT effects on GluN1/2A and GluN1/2B or 2C receptors was the opposite. Whereas currents through GluN1/2A receptors in the second branch of the concentration–activation curve were decreased by DTT, presumably because of the saturating cleavage of disulfide bonds before the HCY action, the currents through GluN1/2B receptors considerably increased so that the concentration–activation curve approached a monophasicness. Consequently, this pronounced increase of currents through GluN1/2B receptors activated by HCY made them be of similar amplitudes to currents activated by NMDA, suggesting that the DTT prevented the HCY desensitization of these receptors. The DTT potentiation of currents through GluN1/2C receptors was moderate and observed at the largest [HCY] used in these experiments.

### 4.3. HCY Redox Modulation of NMDARs Is GluN2 Subunit-Specific

To understand how redox regulation by HCY may contribute to the functioning of a mixture of NMDARs of different subunit compositions, pharmacological experiments with the usage of GluN2B and GluN2A selective antagonists, which are ifenprodil and Zn^2+^, correspondently, were performed on native NMDARs of cortical neurons. We demonstrated that currents activated by 100 µM HCY at the steady state could be suppressed by GluN2A-selective, but not GluN2B-selective antagonist, that exhibits a lack of contribution of GluN2B-containing receptors to the currents, presumably because of their desensitization. Currents activated by 1000 µM HCY at the steady state, however, were effectively inhibited by the GluN2B-selective antagonist. The remarkable increase of an involvement of GluN2B-containing NMDARs to the ion transfer during HCY-activated responses most likely occurred due to an absence of GluN1/2B receptor desensitization abolished by self-redox regulation by 1000 µM HCY. In fact, the elimination of HCY-induced desensitization of GluN2B-containing NMDARs was achieved by redox modulation with both 1 mM DTT and 1 mM HCY. Presumably, the transition of GluN2B-containing receptors to the desensitized state after their activation by low [HCY]s (of about 100 μM) captures the HCY molecules on the binding sites, since the further recovery conformational changes are prohibited. The cleavage of disulfide bonds either by 1 mM DTT or HCY makes the NMDAR molecule more flexible, which allows the recovery from desensitization.

The structural basis for GluN2 subunit-specific redox regulation of currents activated by HCY is not yet fully understood. Unlike DTT, the reducing agent H_2_S shows GluN2 subunit-specific modulation of NMDARs [33]. Probably, the effects of both *s*-persulfidation and *s*-homocysteinylation depend on the difference of redox-reactive sites between GluN1/2A and GluN1/2B receptors [31,32,34]. In addition, Bolton et al. [50] described that in the presence of low (about 200 nM) glycine, HCY potentiates glutamate-activated NMDAR currents interacting as an agonist with the glycine binding sites. This effect disappeared in the presence of 10 µM glycine. Therefore, it seems unlikely that it could contribute to our observations because the saturating concentration of glycine (30 µM) was used in our experiments.

It is now commonly accepted that GluN2A-dependent accumulation of free intracellular Ca^2+^ is responsible for HCY-induced neurotoxicity [51,52]. We have found that cleaving NMDAR disulfide bonds forces HCY-induced Ca^2+^ accumulation to depend on GluN2B, but not GluN2A subunit-containing NMDARs. The approximate concentration range for HCY redox effects strongly exceeds normal and mild hyperhomocysteinemia levels but could be achieved in severe hyperhomocysteinemia ([HCY] > 200 µM).

Cortical neurons express both GluN2A and GluN2B NMDAR subunits [38,39,40]. The reduction of disulfide bonds caused the opposite effects on these receptor subtypes. However, HCY action on triheteromeric GluN1/2A/2B receptors widely expressed in hippocampal [53] and cortical [41] synapses is still unknown and requires further study.

## 5. Conclusions

The thiol-containing agents like HCY can cleave disulfide bonds at redox-sensitive sites of NMDARs, and the efficacy of HCY as a reducing agent augments with the [HCY] increase. The biphasic concentration–activation relationship of NMDAR currents allows the distinction between the HCY agonist effect and the HCY-positive redox modulation. An estimated concentration for HCY redox effects exceeds mild hyperhomocysteinemia levels but could be achieved in severe hyperhomocysteinemia (>200 µM). We demonstrated that the cleavage of disulfide bonds in the NMDAR molecule enhances HCY-activated transmembrane currents due to reduced desensitization of GluN1/2B receptors. Consequently, cleaving NMDAR disulfide bonds makes HCY-induced Ca^2+^ accumulation dependent on GluN2B, rather than GluN2A subunit-containing NMDARs.

## Abbreviations

[Ca^2+^]	free intracellular calcium concentration
DTT	dithiothreitol
EC_50_	half-maximal effective concentration
GluN1, GluN2A, GluN2B, and GluN2C	subunits of *N*-methyl-d-aspartate receptors
GSH	glutathione
HCY	homocysteine
[HCY]	homocysteine concentration
H_2_S	hydrogen sulfide
Ifen	ifenprodil
NMDAR	*N*-methyl-d-aspartate receptor
SEM	standard error of mean
V_h_	membrane holding potential
